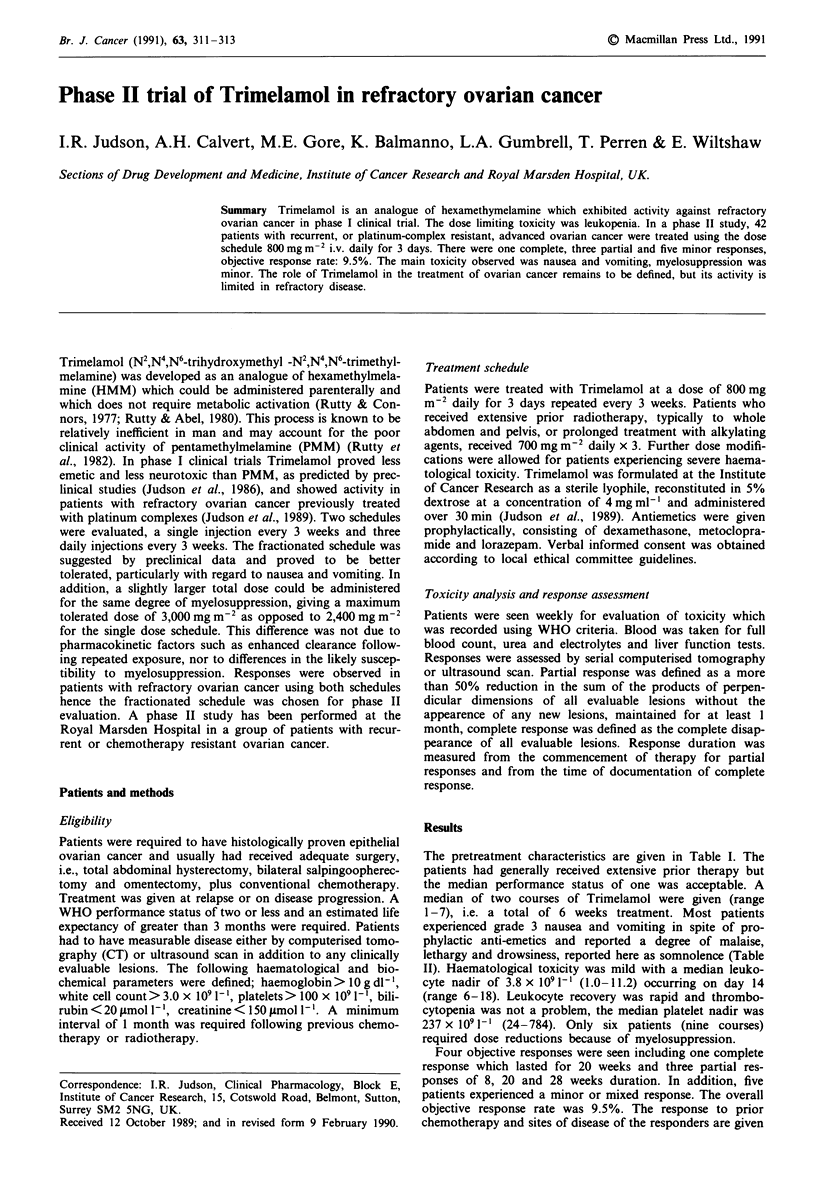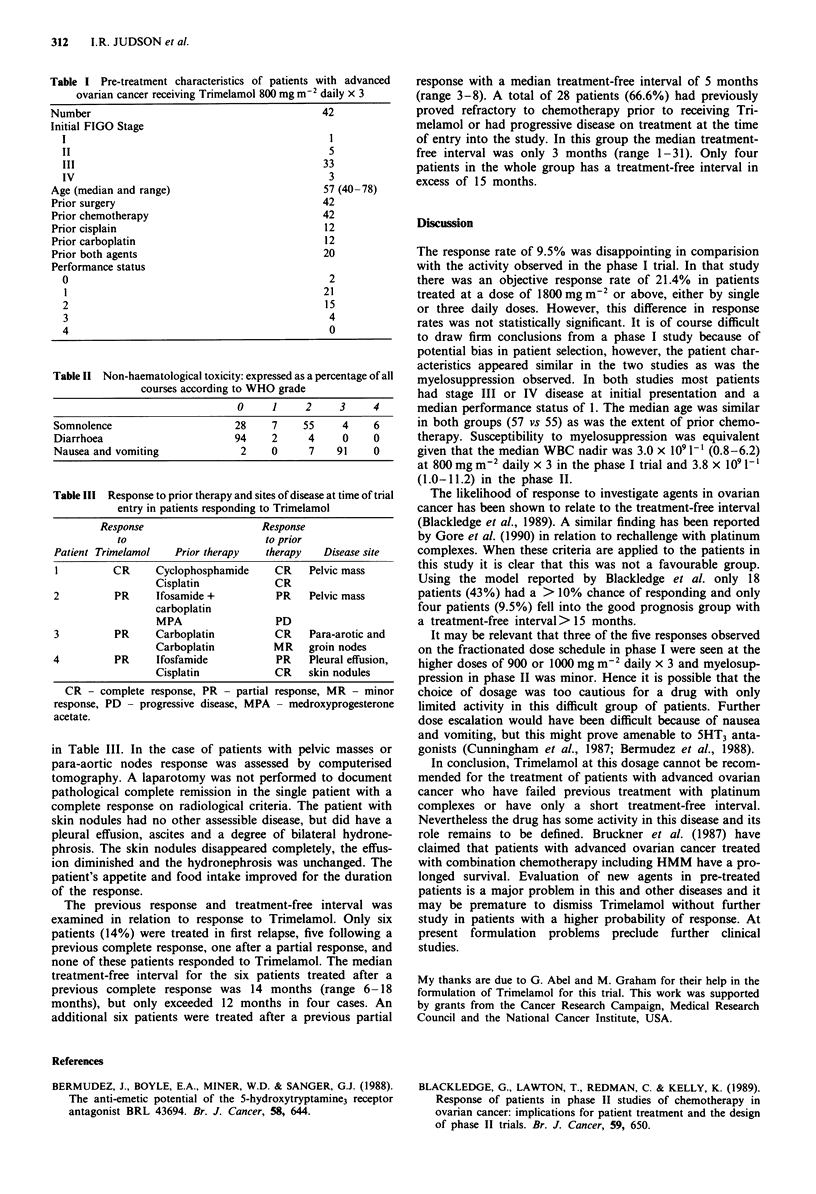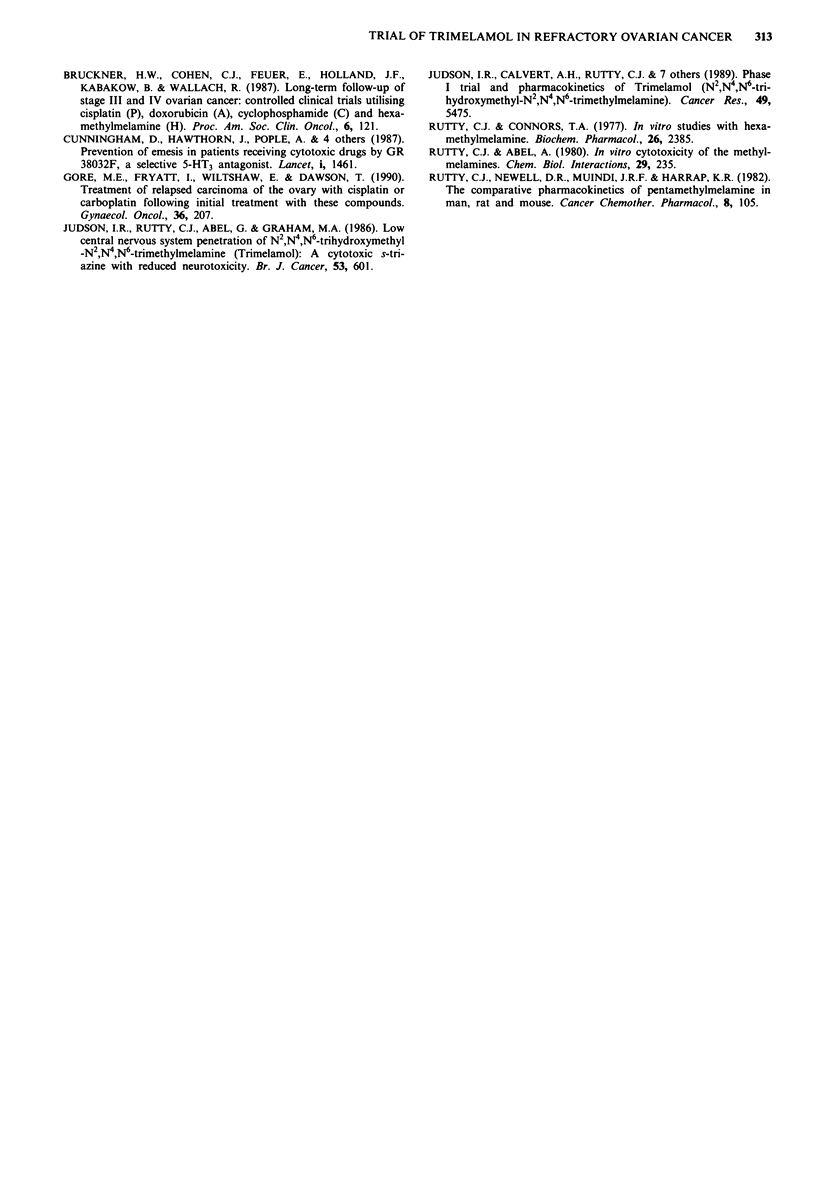# Phase II trial of trimelamol in refractory ovarian cancer.

**DOI:** 10.1038/bjc.1991.72

**Published:** 1991-02

**Authors:** I. R. Judson, A. H. Calvert, M. E. Gore, K. Balmanno, L. A. Gumbrell, T. Perren, E. Wiltshaw

**Affiliations:** Section of Drug Development, Institute of Cancer Research, Royal Marsden Hospital, UK.

## Abstract

Trimelamol is an analogue of hexamethymelamine which exhibited activity against refractory ovarian cancer in phase I clinical trial. The dose limiting toxicity was leukopenia. In a phase II study, 42 patients with recurrent, or platinum-complex resistant, advanced ovarian cancer were treated using the dose schedule 800 mg m-2 i.v. daily for 3 days. There were one complete, three partial and five minor responses, objective response rate: 9.5%. The main toxicity observed was nausea and vomiting, myelosuppression was minor. The role of Trimelamol in the treatment of ovarian cancer remains to be defined, but its activity is limited in refractory disease.


Br. J. Cancer (1991), 63, 311–313 © Macmillan Press Ltd., 1991

### Phase II trial of Trimelamol in refractory ovarian cancer

I.R. Judson, A.H. Calvert, M.E. Gore, K. Balmanno, L.A. Gumbrell, T. Perren & E. Wiltshaw

*Sections of Drug Development and Medicine, Institute of Cancer Research and Royal Marsden Hospital, UK.*

**Summary** Trimelamol is an analogue of hexamethymelamine which exhibited activity against refractory ovarian cancer in phase I clinical trial. The dose limiting toxicity was leukopenia. In a phase II study, 42 patients with recurrent, or platinum-complex resistant, advanced ovarian cancer were treated using the dose schedule 800 mg $m^{-2}$ i.v. daily for 3 days. There were one complete, three partial and five minor responses, objective response rate: 9.5%. The main toxicity observed was nausea and vomiting, myelosuppression was minor. The role of Trimelamol in the treatment of ovarian cancer remains to be defined, but its activity is limited in refractory disease.

Trimelamol ($N^2$,$N^4$,$N^6$-trihydroxymethyl -$N^2$,$N^4$,$N^6$-trimethylmelamine) was developed as an analogue of hexamethylmelamine (HMM) which could be administered parenterally and which does not require metabolic activation (Rutty & Connors, 1977; Rutty & Abel, 1980). This process is known to be relatively inefficient in man and may account for the poor clinical activity of pentamethylmelamine (PMM) (Rutty *et al.*, 1982). In phase I clinical trials Trimelamol proved less emetic and less neurotoxic than PMM, as predicted by preclinical studies (Judson *et al.*, 1986), and showed activity in patients with refractory ovarian cancer previously treated with platinum complexes (Judson *et al.*, 1989). Two schedules were evaluated, a single injection every 3 weeks and three daily injections every 3 weeks. The fractionated schedule was suggested by preclinical data and proved to be better tolerated, particularly with regard to nausea and vomiting. In addition, a slightly larger total dose could be administered for the same degree of myelosuppression, giving a maximum tolerated dose of 3,000 mg $m^{-2}$ as opposed to 2,400 mg $m^{-2}$ for the single dose schedule. This difference was not due to pharmacokinetic factors such as enhanced clearance following repeated exposure, nor to differences in the likely susceptibility to myelosuppression. Responses were observed in patients with refractory ovarian cancer using both schedules hence the fractionated schedule was chosen for phase II evaluation. A phase II study has been performed at the Royal Marsden Hospital in a group of patients with recurrent or chemotherapy resistant ovarian cancer.

### Patients and methods

### *Eligibility*

Patients were required to have histologically proven epithelial ovarian cancer and usually had received adequate surgery, i.e., total abdominal hysterectomy, bilateral salpingoopherectomy and omentectomy, plus conventional chemotherapy. Treatment was given at relapse or on disease progression. A WHO performance status of two or less and an estimated life expectancy of greater than 3 months were required. Patients had to have measurable disease either by computerised tomography (CT) or ultrasound scan in addition to any clinically evaluable lesions. The following haematological and biochemical parameters were defined; haemoglobin $> 10$ g $dl^{-1}$, white cell count $> 3.0 \times 10^9 l^{-1}$, platelets $> 100 \times 10^9 l^{-1}$, bilirubin $< 20$ μmol $l^{-1}$, creatinine $< 150$ μmol $l^{-1}$. A minimum interval of 1 month was required following previous chemotherapy or radiotherapy.

Correspondence: I.R. Judson, Clinical Pharmacology, Block E, Institute of Cancer Research, 15, Cotswold Road, Belmont, Sutton, Surrey SM2 5NG, UK.

Received 12 October 1989; and in revised form 9 February 1990.

### *Treatment schedule*

Patients were treated with Trimelamol at a dose of 800 mg $m^{-2}$ daily for 3 days repeated every 3 weeks. Patients who received extensive prior radiotherapy, typically to whole abdomen and pelvis, or prolonged treatment with alkylating agents, received 700 mg $m^{-2}$ daily $\times$ 3. Further dose modifications were allowed for patients experiencing severe haematological toxicity. Trimelamol was formulated at the Institute of Cancer Research as a sterile lyophile, reconstituted in 5% dextrose at a concentration of 4 mg $ml^{-1}$ and administered over 30 min (Judson *et al.*, 1989). Antiemetics were given prophylactically, consisting of dexamethasone, metoclopramide and lorazepam. Verbal informed consent was obtained according to local ethical committee guidelines.

### *Toxicity analysis and response assessment*

Patients were seen weekly for evaluation of toxicity which was recorded using WHO criteria. Blood was taken for full blood count, urea and electrolytes and liver function tests. Responses were assessed by serial computerised tomography or ultrasound scan. Partial response was defined as a more than 50% reduction in the sum of the products of perpendicular dimensions of all evaluable lesions without the appearence of any new lesions, maintained for at least 1 month, complete response was defined as the complete disappearance of all evaluable lesions. Response duration was measured from the commencement of therapy for partial responses and from the time of documentation of complete response.

### Results

The pretreatment characteristics are given in Table I. The patients had generally received extensive prior therapy but the median performance status of one was acceptable. A median of two courses of Trimelamol were given (range 1–7), i.e. a total of 6 weeks treatment. Most patients experienced grade 3 nausea and vomiting in spite of prophylactic anti-emetics and reported a degree of malaise, lethargy and drowsiness, reported here as somnolence (Table II). Haematological toxicity was mild with a median leukocyte nadir of $3.8 \times 10^9 l^{-1}$ (1.0–11.2) occurring on day 14 (range 6–18). Leukocyte recovery was rapid and thrombocytopenia was not a problem, the median platelet nadir was $237 \times 10^9 l^{-1}$ (24–784). Only six patients (nine courses) required dose reductions because of myelosuppression.

Four objective responses were seen including one complete response which lasted for 20 weeks and three partial responses of 8, 20 and 28 weeks duration. In addition, five patients experienced a minor or mixed response. The overall objective response rate was 9.5%. The response to prior chemotherapy and sites of disease of the responders are given

**Table I** Pre-treatment characteristics of patients with advanced ovarian cancer receiving Trimelamol 800 mg $m^{-2}$ daily × 3

| | |
|---|---|
| Number | 42 |
| Initial FIGO Stage | |
| I | 1 |
| II | 5 |
| III | 33 |
| IV | 3 |
| Age (median and range) | 57 (40–78) |
| Prior surgery | 42 |
| Prior chemotherapy | 42 |
| Prior cisplain | 12 |
| Prior carboplatin | 12 |
| Prior both agents | 20 |
| Performance status | |
| 0 | 2 |
| 1 | 21 |
| 2 | 15 |
| 3 | 4 |
| 4 | 0 |

**Table II** Non-haematological toxicity: expressed as a percentage of all courses according to WHO grade

| | *0* | *1* | *2* | *3* | *4* |
|---|---|---|---|---|---|
| Somnolence | 28 | 7 | 55 | 4 | 6 |
| Diarrhoea | 94 | 2 | 4 | 0 | 0 |
| Nausea and vomiting | 2 | 0 | 7 | 91 | 0 |

**Table III** Response to prior therapy and sites of disease at time of trial entry in patients responding to Trimelamol

| *Patient* | *Response to Trimelamol* | *Prior therapy* | *Response to prior therapy* | *Disease site* |
|---|---|---|---|---|
| 1 | CR | Cyclophosphamide | CR | Pelvic mass |
| | | Cisplatin | CR | |
| 2 | PR | Ifosamide + carboplatin | PR | Pelvic mass |
| | | MPA | PD | |
| 3 | PR | Carboplatin | CR | Para-arotic and |
| | | Carboplatin | MR | groin nodes |
| 4 | PR | Ifosfamide | PR | Pleural effusion, |
| | | Cisplatin | CR | skin nodules |

CR – complete response, PR – partial response, MR – minor response, PD – progressive disease, MPA – medroxyprogesterone acetate.

in Table III. In the case of patients with pelvic masses or para-aortic nodes response was assessed by computerised tomography. A laparotomy was not performed to document pathological complete remission in the single patient with a complete response on radiological criteria. The patient with skin nodules had no other assessible disease, but did have a pleural effusion, ascites and a degree of bilateral hydronephrosis. The skin nodules disappeared completely, the effusion diminished and the hydronephrosis was unchanged. The patient's appetite and food intake improved for the duration of the response.

The previous response and treatment-free interval was examined in relation to response to Trimelamol. Only six patients (14%) were treated in first relapse, five following a previous complete response, one after a partial response, and none of these patients responded to Trimelamol. The median treatment-free interval for the six patients treated after a previous complete response was 14 months (range 6–18 months), but only exceeded 12 months in four cases. An additional six patients were treated after a previous partial response with a median treatment-free interval of 5 months (range 3–8). A total of 28 patients (66.6%) had previously proved refractory to chemotherapy prior to receiving Trimelamol or had progressive disease on treatment at the time of entry into the study. In this group the median treatment-free interval was only 3 months (range 1–31). Only four patients in the whole group has a treatment-free interval in excess of 15 months.

### Discussion

The response rate of 9.5% was disappointing in comparision with the activity observed in the phase I trial. In that study there was an objective response rate of 21.4% in patients treated at a dose of 1800 mg $m^{-2}$ or above, either by single or three daily doses. However, this difference in response rates was not statistically significant. It is of course difficult to draw firm conclusions from a phase I study because of potential bias in patient selection, however, the patient characteristics appeared similar in the two studies as was the myelosuppression observed. In both studies most patients had stage III or IV disease at initial presentation and a median performance status of 1. The median age was similar in both groups (57 *vs* 55) as was the extent of prior chemotherapy. Susceptibility to myelosuppression was equivalent given that the median WBC nadir was $3.0 \times 10^9 l^{-1}$ (0.8–6.2) at 800 mg $m^{-2}$ daily × 3 in the phase I trial and $3.8 \times 10^9 l^{-1}$ (1.0–11.2) in the phase II.

The likelihood of response to investigate agents in ovarian cancer has been shown to relate to the treatment-free interval (Blackledge *et al.*, 1989). A similar finding has been reported by Gore *et al.* (1990) in relation to rechallenge with platinum complexes. When these criteria are applied to the patients in this study it is clear that this was not a favourable group. Using the model reported by Blackledge *et al.* only 18 patients (43%) had a $>10\%$ chance of responding and only four patients (9.5%) fell into the good prognosis group with a treatment-free interval $>15$ months.

It may be relevant that three of the five responses observed on the fractionated dose schedule in phase I were seen at the higher doses of 900 or 1000 mg $m^{-2}$ daily × 3 and myelosuppression in phase II was minor. Hence it is possible that the choice of dosage was too cautious for a drug with only limited activity in this difficult group of patients. Further dose escalation would have been difficult because of nausea and vomiting, but this might prove amenable to $5HT_3$ antagonists (Cunningham *et al.*, 1987; Bermudez *et al.*, 1988).

In conclusion, Trimelamol at this dosage cannot be recommended for the treatment of patients with advanced ovarian cancer who have failed previous treatment with platinum complexes or have only a short treatment-free interval. Nevertheless the drug has some activity in this disease and its role remains to be defined. Bruckner *et al.* (1987) have claimed that patients with advanced ovarian cancer treated with combination chemotherapy including HMM have a prolonged survival. Evaluation of new agents in pre-treated patients is a major problem in this and other diseases and it may be premature to dismiss Trimelamol without further study in patients with a higher probability of response. At present formulation problems preclude further clinical studies.

My thanks are due to G. Abel and M. Graham for their help in the formulation of Trimelamol for this trial. This work was supported by grants from the Cancer Research Campaign, Medical Research Council and the National Cancer Institute, USA.

### References

BERMUDEZ, J., BOYLE, E.A., MINER, W.D. & SANGER, G.J. (1988). The anti-emetic potential of the 5-hydroxytryptamine$_3$ receptor antagonist BRL 43694. *Br. J. Cancer*, **58**, 644.

BLACKLEDGE, G., LAWTON, T., REDMAN, C. & KELLY, K. (1989). Response of patients in phase II studies of chemotherapy in ovarian cancer: implications for patient treatment and the design of phase II trials. *Br. J. Cancer*, **59**, 650.

BRUCKNER, H.W., COHEN, C.J., FEUER, E., HOLLAND, J.F., KABAKOW, B. & WALLACH, R. (1987). Long-term follow-up of stage III and IV ovarian cancer: controlled clinical trials utilising cisplatin (P), doxorubicin (A), cyclophosphamide (C) and hexamethylmelamine (H). *Proc. Am. Soc. Clin. Oncol.*, **6**, 121.

CUNNINGHAM, D., HAWTHORN, J., POPLE, A. & 4 others (1987). Prevention of emesis in patients receiving cytotoxic drugs by GR 38032F, a selective 5-$HT_3$ antagonist. *Lancet*, **i**, 1461.

GORE, M.E., FRYATT, I., WILTSHAW, E. & DAWSON, T. (1990). Treatment of relapsed carcinoma of the ovary with cisplatin or carboplatin following initial treatment with these compounds. *Gynaecol. Oncol.*, **36**, 207.

JUDSON, I.R., RUTTY, C.J., ABEL, G. & GRAHAM, M.A. (1986). Low central nervous system penetration of $N^2$,$N^4$,$N^6$-trihydroxymethyl -$N^2$,$N^4$,$N^6$-trimethylmelamine (Trimelamol): A cytotoxic *s*-triazine with reduced neurotoxicity. *Br. J. Cancer*, **53**, 601.

JUDSON, I.R., CALVERT, A.H., RUTTY, C.J. & 7 others (1989). Phase I trial and pharmacokinetics of Trimelamol ($N^2$,$N^4$,$N^6$-trihydroxymethyl-$N^2$,$N^4$,$N^6$-trimethylmelamine). *Cancer Res.*, **49**, 5475.

RUTTY, C.J. & CONNORS, T.A. (1977). *In vitro* studies with hexamethylmelamine. *Biochem. Pharmacol.*, **26**, 2385.

RUTTY, C.J. & ABEL, A. (1980). *In vitro* cytotoxicity of the methylmelamines. *Chem. Biol. Interactions*, **29**, 235.

RUTTY, C.J., NEWELL, D.R., MUINDI, J.R.F. & HARRAP, K.R. (1982). The comparative pharmacokinetics of pentamethylmelamine in man, rat and mouse. *Cancer Chemother. Pharmacol.*, **8**, 105.